# Reaching Patients With Noncommunicable Diseases in Rural Tanzania Using Mobile Devices and Community Trust: Qualitative Study

**DOI:** 10.2196/29407

**Published:** 2022-03-17

**Authors:** Ayano Miyashita, Keiko Nakamura, Mayumi Ohnishi, Deogratius Bintabara, Festo K Shayo, Isaac I Maro, Hideko Sato, Kaoruko Seino, Stephen Kibusi

**Affiliations:** 1 Department of Global Health Entrepreneurship Tokyo Medical and Dental University Tokyo Japan; 2 Department of Community-Based Rehabilitation Sciences Nagasaki University Nagasaki Japan; 3 College of Health Sciences The University of Dodoma Dodoma United Republic of Tanzania; 4 The Ifakara Health Institute Dar es Salaam United Republic of Tanzania

**Keywords:** noncommunicable disease, community health workers, Tanzania, communication, rural, community, trust, disease, acceptability, usability, text message, SMS, mobile phone, implementation

## Abstract

**Background:**

A health service using mobile devices, mobile health (mHealth), has been widely applied to programs focusing on maternal and child health and communicable diseases in sub-Saharan African countries. However, mHealth apps for noncommunicable disease (NCD) services remain limited.

**Objective:**

This study aimed to explore the acceptability and potential usability of SMS text messaging for patients and health care providers for the management of NCDs as part of an implementation research in rural Tanzania.

**Methods:**

Nine focus group discussions were conducted with 56 participants (21 community health workers [CHWs], 17 patients, and 18 health care professionals [HPs]) in 3 districts in the Dodoma region, Tanzania. The interview guides were prepared in Swahili, and each session was recorded, transcribed, and translated into English. The focus group discussions consisted of the following topics: (1) perceptions of the participants about the possible use of mobile devices and SMS text messages as an mHealth platform in community health services; and (2) experiences of mobile device use in health activities or receiving health services via a mobile phone in the past.

**Results:**

CHWs and HPs reported having familiarity using mobile devices to provide health services, especially for reaching or tracing patients in remote settings; however, patients with NCDs were less familiar with the use of mobile devices compared with the other groups. Hesitation to receive health services via SMS text messaging was seen in the patient group, as they wondered who would send health advice to them. Some patients expected services beyond what mHealth could do, such as aiding in recovery from a disease or sending notifications about the availability of prescription medications. CHWs showed interest in using text messaging to provide health services in the community; however, the concerns raised by CHWs included the cost of using their own mobile devices. Moreover, they demanded training about NCD management before engaging in such an activity.

**Conclusions:**

This study explored views and experiences regarding the possible installation of an mHealth intervention for managing NCDs in rural Tanzania. Although HPs and CHWs had experience using mobile devices to provide health services in non-NCD projects, only a few patients (3/17, 17%) had heard about the use of mobile devices to receive health services. To improve the suitability and acceptability of the intervention design for patients with NCDs, their trust must be earned. Involving CHWs in the intervention is recommended because they have already been appointed in the community and already know how to communicate effectively with patients in the area.

## Introduction

In 2011, mobile health (mHealth) was defined as “the use of mobile and wireless technologies to support the achievement of health objectives” by the World Health Organization [1] and is expected to be an increasingly important tool for patients in resource-scarce settings due to the fast-growing penetration rates of mobile phones in developing countries [2-4]. mHealth has already been implemented for a wide range of health promotion and management activities: to act as reminders of clinic visits, to encourage medication adherence, and to promote behavioral changes [5,6]. The increase in the numbers of patients with noncommunicable disease (NCDs) is likely to dominate health care needs and expenditures in most low-income and middle-income countries [7]. Tanzania is no exception and is facing an increasing prevalence of NCDs at a time when its health system is already overloaded with a high prevalence of communicable diseases [8,9]. A national survey in 2012 showed that the prevalence of Diabetes Mellitus (DM) and hypertension (HT) was found to be 9.1% and 25.9%, respectively [10]. In 2016, there were approximately 0.14 physicians per 10,000 individuals nationwide, with a lower ratio in the rural areas [11]. The Tanzania Ministry of Health’s strategic and action plan for the prevention and control of NCDs is targeting to reduce mortality by 25% from baseline by 2025 by improving patients’ disease management through community and facility care interventions [12]. Although the country has a well-thought-out NCD strategic plan, the already overburdened health system is limiting the effective control of NCDs. mHealth, which uses text messages in Tanzania, has been applied only in programs to combat communicable diseases and reproductive health issues. This study is a part of the Community mHealth Integrated Care (ComHIC) to manage hypertension (HT) or diabetes (DM) in Tanzania’s overburdened health system [13]. Our project aims to apply mHealth with support by community health workers (CHWs) under an NCD program to overcome the lack of resources and infrastructure for health service access in the rural Dodoma districts. Our mHealth system is aiming to support the existing local health systems to help control and manage patients with HT and DM. The objectives of this study were to obtain insights into perceptions of the concept of mHealth among CHWs, patients with NCDs, and health care professionals (HPs) in rural Tanzania, to assess the possible use of mobile devices and SMS text messaging as an mHealth platform among CHWs and patients, and to develop an appropriate mHealth intervention for the study site.

## Methods

### Setting and Research Team

Focus group discussions (FGDs) were conducted in the Kondoa, Kongwa, and Mpwapwa districts in the Dodoma region of Tanzania from January to February 2020. The research team consists of health system researchers, health literacy and communication experts with experience of qualitative data collection, physicians, and nurses with experiences in Tanzania, who developed a protocol of the study.

### Eligibility Criteria of the Participants

Quota sampling method was applied to recruit 3 participant groups of CHWs, patients with diabetes or hypertension, and HPs at each district hospital, a total of 9 groups with 6-8 people in each group. A Dodoma regional medical officer gave a recruiting instruction to NCD coordinators who work for the NCD clinics at each district hospital, and they called in the potential candidates. For the 3 groups, the candidates were listed based on the following eligibility criteria: for all—no gender quota; aged above 18 years; and willing and capable to attend a 60-minute interview session held at the district hospital; for CHWs—those who have registered at the district hospitals and therefore have been engaging in a health program or have worked as a CHW in the past; for patients—attending NCD clinics at the district hospital more than 1 year for HT, DM, or both; and for HPs—those who are currently working at NCD clinics at the district hospitals.

### Interview Guides Development

The 3 interview guides for CHWs, for patients with DM or HT, and for HPs were prepared in Swahili by the study team through a series of discussions among the study team. A semistructured interview design was adopted with consultations with researchers who had experience in designing interview guides. The main topics of the discussions were (1) perceptions of the participants about the possible use of mobile devices and SMS text messaging as an mHealth platform in community health services; and (2) experiences of mobile device use in health activities or receiving health services via a mobile phone in the past.

### Qualitative Interview and Analysis

A facilitator and a notetaker, both native Swahili speakers, moderated the FGD sessions. Prior to the initiation of the FGDs, the facilitator gave a brief explanation of the study purpose and obtained written informed consent from all participants who had agreed to participate. All FGDs were conducted in a room at the district hospital. The FGDs, which were approximately 60 minutes per session, were audio recorded, transcribed, and translated into English. The transcripts were then uploaded to NVivo, version 12 (QSR International) [14], and the analysis was performed inductively. The initial sets of code were extracted by 2 investigators individually, and they were compared to each other to increase transparency in the process of code and category generation and to ensure consistency in the codes. Any inconsistencies or questions that arose during the individual analysis were recorded and discussed between the 2 investigators. When such issues could not be resolved by comparing the generated sets of code, the investigators referred to the original texts and interviewed the researcher members who conducted the interviews. Those processes of reexamination were repeated until the final analysis results were obtained. Further, the results were reviewed by the study team including members who facilitated the focus group interviews and the physicians and nurses who were familiar with the regional context for the analyses to capture the participants’ perspectives.

### Ethics

Ethical clearance certificate for conducting medical research in Tanzania was obtained from the National Institute for Medical Research (HQ/R.8a/Vol.IX/3220). The study was also approved by the Research Review Committee of Tokyo Medical and Dental University, Japan (M2019-191).

## Results

### Participants

Nine FGDs were conducted with a total of 21 (38%) CHWs, 17 (30%) patients, and 18 (32%) HPs (n=56; 22 men [39%], 34 women [61%]) in the 3 districts in the Dodoma region. The age ranges of the participants were 24-61 years for CHWs, 27-54 years for HPs, and 55-68 years for patients; however, 11 (65%) of the 17 patients did not agree to provide their date of birth, as some believed the information could be used for witchcraft. Multimedia Appendix 1 shows the participants’ perspectives and experiences regarding the use of mHealth. The results of the discussion are described as follows.

### Perceptions of mHealth

The participants’ perceptions about the possible use of mobile devices and SMS text messaging as an mHealth platform in community health services are as follows:

#### Positive Perceptions

All groups provided essentially positive feedback about the use of mHealth for supporting NCD control and management. CHWs supported the idea of using SMS text messaging to contact patients for several reasons. The main reason was saving time and resources, as mHealth enables them to contact many patients in a limited time, to overcome transportation issues, and to save time reporting. Possible mobile uses for NCD control included providing health information, offering encouragement, and sending reminders for clinical appointments and taking medications.

I see this is a simple way of communicating with them because you cannot visit them all; you may find that you have eighty or ninety patients; you cannot visit all of them; so mobile phones are the easiest means.

CHW, Kondoa

The majority of patients (4/5, 80% in Kondoa; 5/6, 83% in Kongwa; and 5/6, 83% in Mpwapwa) agreed about the use of mobile devices for reminders for clinical appointments and medications. All FGD participants were confident about reading SMS text messages and using mobile devices for communication. Some patients were willing to follow treatment guidelines if they were provided with one via mobile, while others said that regular SMS text messages would make them feel encouraged and supported.

It’s a good idea, everybody knows how to read a message here. It is readable, and I think more beautiful.

Patient, Mpwapwa

HPs agreed about the use of SMS text messages to support patients with NCD, as they believe the majority of people currently have mobile devices, and they have seen some successful health projects using mobile devices in the past. They think that messages can motivate patients to follow their recommendations and reduce the number of missed appointments; therefore, they hope the intervention could lead to a reduced burden of complications, blood pressure, and blood sugar. Some believe that emotional support for patients with long-term health issues could be provided by text because some patients have been described as feeling lonely.

I think it’s a good idea. We have a habit that everyone likes to be reminded of things, and for most patients, especially those with loneliness diseases, a little reminder makes him/her know that you do care.

HP, Kongwa

The use of mHealth as an educational tool was also expected, and reaching patients who do not know how to read or have a mobile device was suggested via family members.

If I try to look at the kind of patients we see, most are old, and these old people are over sixty years of age; most of them don’t use mobile phones on a large scale, and even if they do, they maybe just receive a call from their children. But many have difficulty reading; so, the use of mobile phones may not necessarily help to reach them directly. We can reach them through their children and grandchildren and explain things to them as they receive the text message.

HP, Kondoa

#### Negative Perceptions

As CHWs had already been using their mobile devices to reach out to patients for the current project, they were also aware of the possible difficulties that may arise. They mentioned that not all patients have a mobile device; however, they said it was possible to reach patients via a mobile device belonging to a relative or someone the patients could trust. Their biggest concern was how the project provides funds to purchase airtime vouchers, as many of them cover the cost out-of-pocket.

*To add to that, it is our routine/arrangement that every patient must provide a mobile phone number; if he/she doesn’t have a number, then they provide the number of aclose relative to whom he/she trusts to share his/her health problem. We have established this system so that we can reach our clients; otherwise, we would lose many*. [CHW, Kondoa]

Some patients had never heard the term NCDs, so they were not very sure about the content of the texts and from whom they would receive them. Especially in Mpwapwa, the concept of mHealth was not easily grasped during the interview.

I see the question… but maybe the question was, I did not really understand, if I ever received any messages on the phone about these diseases we are talking about, diabetes and hypertension? I said I never did, except if it is health care, this the system that the whole of Tanzania should use to be treated.

Patient, Mpwapwa

Really good advice, now how do they get my phone number? And who will send me such a message? You or someone else? He has to, he doesn't have my phone number, I don't know him, how will he serve me?

Patient, Mpwapwa

Although the majority of the HPs provided positive feedback about mHealth, some shared concerns that the use of mHealth only may not be sufficient as an intervention. They suggested combining other types of health promotion such as TV, radio, billboards, and social networking services to not only cover patients who are illiterate or do not have a mobile device, but also to promote health in the general population.

Yes, it can help a lot for those who have access to cell phones because not all of them have phones or they have them but they cannot use them; he/she can answer a call but cannot read a text message. Let’s not just use the cell phones; also, posters like that big one at the bus terminal with a message “TB is preventable.” It can also be done through TV because many watch TV.

HP, Kondoa

Other concerns were related to difficulties eliciting behavioral change, general negligence toward interventions, and a lack of awareness leading to misperceptions.

#### Recommendations or Expectations for mHealth for Each Participant Group

CHWs expect the project to provide airtime vouchers for communicating with patients. In addition, some hope to have a device such as tablet or smartphone for reporting their activities. Regarding the mode of communication, in general, CHWs prefer two-way communication.

My opinions are: we suggest making it possible to be funded for airtime vouchers so that we can communicate with our clients. Most of them have phones, only a few of them do not. If we would be given airtime vouchers, we could contact our clients anytime we needed to.

CHW, Kongwa

Once patients understood the mHealth concept, reminders for clinical appointments and taking medications were popular functions they hoped to have. Some described that they were expecting to receive medical advice via their mobile device, while others expected to see clinical outcomes such as decreased blood pressure or alleviated complications after following the obtained advice. Most preferred receiving short messages, except some, who preferred calls instead of texts for medication reminders. Expectations of being able to order prescribed medications and receive notifications about medication supplies were also reported.

I really thought it would be better because sometimes you can forget the time to take a medicine; but if we get a call to remind us, it will be better for me.

Patient, Kongwa

HPs suggested SMS text message contents such as giving reminders of clinical appointments and medication times, promoting dietary salt reduction and exercise, and advising about lifestyle modifications. Regarding strategies for the effective delivery of messages to patients, concrete advice such as sending a series of short, well-written messages at least once a week (the time of the day depending on the disease) and risk communication, including the impact of complications and the economic burden for families, were also raised during the interviews.

There are reminders that could be sent on lifestyle modifications and adherence to medications, so these could be just short messages, but specific, such as only food intake or exercise, not just one message that has everything in it.

HP, Mpwapwa

### Experiences With mHealth

Here, we turn to the experiences of mobile device use in health activities or receiving health services via a mobile phone in the past. FGDs included participant critiques of using mobiles, which are as follows:

1. CHWs reported that at least 5 organizations were using some sort of communication tools, including those for maternal and child health, family planning, human immunodeficiency virus infection and acquired immunodeficiency syndrome (HIV/AIDS), and tuberculosis. Some nongovernmental organizations provide a tablet to every CHW so that they can write a monthly report.
2. Patients in the Mpwapwa district were not aware of any projects using mobile devices. One of the patients in the Kongwa district mentioned the use of mobile devices for reminders to patients about clinical visits at a referral hospital. Two patients in Kondoa reported hearing about HIV/AIDS projects that use mobile devices to contact patients for checking and tracing (3/17, 17% patients).
3. HPs reported knowing about various projects that were using mHealth for adherence support and follow-up for patients, such as those for maternal and child health, family planning, tuberculosis, and leprosy. Apparently, mobile devices were not the only mode of communication; for example, radios were used for a tuberculosis project and billboards for an HIV project. HPs also shared the lessons learned from past projects; for instance, messages that were too long to read were often ignored by the receivers, and inconsistent content demoralized participants in terms of motivation.

## Discussion

Regarding the extent of the brief explanation of mHealth during the interviews, the idea of text-based interventions for NCD management was positively accepted by the CHWs, patients, and HPs. However, some patients had difficulty understanding the concept. Hesitation (eg, who sends the messages?) and unrealistically high expectations (eg, it may fix all problems) about mHealth’s effectiveness were reported. Positive reasoning was based on the commonality of mobile device use for daily activities and geographical hardships experienced during health-seeking and health-providing behaviors. CHWs and HPs supported the use of mobile devices as they had already been using them to reach out to their patients. Even if those targeted among the older population cannot read or do not have a mobile device, CHWs and HPs believed that their children or grandchildren could read out the texts for them.

According to the 2012 STEP (World Health Organization Stepwise Approach to NCD Risk Factor Surveillance) survey in Tanzania, the prevalence of diabetes for men increased with age and reached at peak at the age group of 45-54 while the prevalence for women was high at the age group of 55-64. In the same survey, the prevalence of both diastolic and systolic blood pressure increased with age, and it reached peak level at the age group of 55-64 years [10]. This study covered the major age range of the DM and HT patients in Tanzania. mHealth interventions must be accessible to patients with NCD in rural Dodoma, and if SMS text messages are used, they must be effective and deemed culturally appropriate in order to manage the disease. For example, based on observations made by the facilitator during the focus groups, the research team interpreted different levels of understanding for the mHealth concept among patients across 3 districts. Dialects and cultural diversity were reported within the region. A CHW suggested a combination of SMS text messaging and supports by CHWs; this would facilitate patients’ management of disease and lead to culturally appropriate understanding by local patients. The mHealth intervention program would ideally be implemented locally in terms of consistency and sustainability.

Although the utility of text messages was positively accepted among patients, preferably, the sender of the text messages should acquire community trust in advance to disseminate the NCD management information. HPs and CHWs shared some concerns that a text-only approach may not be sufficient to elicit behavioral change among patients. CHWs stated that they would also like to continue making phone calls, as calling may be easier than typing text messages (although our intervention design did not ask CHWs to prepare text messages), and patients’ understanding can be checked immediately. HPs preferred to combine text messages with other means of health promotion, such as billboards, TV, or radio, since they thought those means cover more people and offer more preventive effects.

The involvement of CHWs in the program could be helpful to reinforce the outcomes of text-based learning or reminders for the patients. CHWs were appointed by a chairperson in village meetings, and they know how to authorize their activity in each community. As a result, they could connect the community (patients) with health care facilities (HPs). CHWs’ knowledge about how to operate health projects in socially tight communities could be valuable to acquire community trust and achieve effective communication with patients. Involving experienced CHWs in the mHealth intervention may help to improve the implementation fidelity of the project.

To achieve the above, training for CHWs about managing patients with NCDs is necessary. Many CHWs have expressed uncertainty regarding knowledge about NCDs, and if there were such a program, they would like to undergo the necessary training before joining. Some concerns were also raised by CHWs such as the cost of using their own mobile device when working for patients, which they must do in current programs. To improve the acceptability and sustainability of the intervention, it is important to minimize the cost of using the intervention for CHWs.

## Appendix

Multimedia Appendix 1 Perspectives of the participants in the focus group discussion.

## Abbreviations

CHW: community health worker
ComHIC: Community mHealth Integrated Care
DM: Diabetes Mellitus
FGD: focus group discussion
HP: health care professional
HT: hypertension
mHealth: mobile health
NCD: noncommunicable disease
STEP: World Health Organization Stepwise Approach to NCD Risk Factor Surveillance
