# Supplementary material for: Reaching Patients With Noncommunicable Diseases in Rural Tanzania Using Mobile Devices and Community Trust: Qualitative Study
Source: JMIR Mhealth Uhealth. 2022 Mar 17;10(3):e29407. doi: 10.2196/29407 (PMC8972119; doi:10.2196/29407)
Supplement: Multimedia Appendix 1 [file mhealth_v10i3e29407_app1.pdf]

Multimedia Appendix 1: Perspectives of the participants in the focus group discussion

[a] Perceptions of the participants about the possible use of mobile devices and SMS text messages as an mHealth platform in community health services

1. Mobile phone text messages can support disease management
[Community health workers (CHWs)]
- Text messages are useful (most people have phones/encourage clients to go to the clinic/adherence support/information provision/encouragement)
- Save time and resources
- Overcome transport issues
- Enable us to communicate with many patients as the number of NCDs increases rapidly
- Save time reporting back to the organization
- Able to check the client's condition
- Simple and reachable communication tool
- Able to contact clients at any time

[Patients]
- Text messages are useful as a reminder (for appointment dates/medication/text messages are better than the current situation)
- Everyone knows how to read a message
- Can use our phone to communicate
- Reminder messages will help improve health (messages will contribute to paying more attention to health/better medication adherence)
- Can follow text guidelines
- Encouragement (feeling supported)

[Healthcare professionals (HPs)]
- Text messages are useful
- Many people use cell phones
- Experience with other programs using text messages in the past
- Messages motivate patients (able to reduce missed appointments/the burden of complications/other chronic diseases)
- Emotional support
- Can be an educational tool (health promotion/reduce disease burden/self-management/improved adherence)
- Text messages may contribute to a reduction in blood pressure/blood sugar

2. Possible opportunities despite challenges
[CHWs]
- Even if patients do not have a mobile phone, a system is already in place to reach them via relatives or someone they trust
- Some CHWs experience difficulties in reaching out to patients during the farming season, but mobile phones may help this situation
- CHWs already know the authorization process in the community via the chairperson

[Patients]
- Patients without a mobile phone can be reached via relatives or someone they trust
- Never heard of using text messages for NCDs
- Knowledge regarding NCDs is low, but they are open to receive information via text messages
- Default tracing by using mobile phones

[HPs]
- Not all HPs have a mobile phone
- Some HPs cannot read, but can involve family members through reading aloud for the patient (if the patient does not have a mobile phone/cannot read)
- May decrease the family burden
- Lack of financial and transportation support at NCD clinics
- Geographical locations are very complex
- Regional disparities

[b] Experiences of mobile device use in health activities or receiving health services via a mobile phone in the past

1. Other programs using text messages in the past
[CHWs]
- Nelson Mandela project (Maternal and Child Health (MCH))
- Mjenga project (contraception)
- Management and Development Health (tuberculosis)
- EngenderHealth (Family Planning-HIV) (four in Kongwa)
- Christian Association in Tanzania: a brave woman in Dodoma region (in Mpwapwa) provided tablets for reporting

[Patients]
- Aware of existing programs
- HIV/AIDS (two in Kondoa)
- Benjamine Mkapa (one in Kongwa)

[HPs]
- Tuberculosis/leprosy
- Adherence support
- MCH: Safe delivery project
- Patient alert system was a success (many turned out)
- Short messages preferred by patients
- Repetition interval maybe necessary

2. Possible opportunities despite challenges
[CHWs]
- Even if patients do not have a mobile phone, a system is already in place to reach them via relatives or someone they trust
- Some CHWs experience difficulties in reaching out to patients during the farming season, but mobile phones may help this situation
- CHWs already know the authorization process in the community via the chairperson

[Patients]
- Patients without a mobile phone can be reached via relatives or someone they trust
- Never heard of using text messages for NCDs
- Knowledge regarding NCDs is low, but they are open to receive information via text messages
- Default tracing by using mobile phones

[HPs]
- Not all HPs have a mobile phone
- Some HPs cannot read, but can involve family members through reading aloud for the patient (if the patient does not have a mobile phone/cannot read)
- May decrease the family burden
- Lack of financial and transportation support at NCD clinics
- Geographical locations are very complex
- Regional disparities
